# 8-Hydroxy-2-Anilino-1,4-Naphthoquinone Prevents Against Ferroptotic Neuronal Death and Kainate-Induced Epileptic Seizures

**DOI:** 10.3390/pharmaceutics17111415

**Published:** 2025-10-31

**Authors:** Daseul Lee, Eun Jung Na, Yumi Heo, Jinha Yu, Hwa-Jung Kim

**Affiliations:** College of Pharmacy and Graduate School of Pharmaceutical Sciences, Ewha Womans University, Seoul 03760, Republic of Korea; dsleee@ewha.ac.kr (D.L.); ejna@ewha.ac.kr (E.J.N.); ymheo0710@ewhain.net (Y.H.); jhyu@ewha.ac.kr (J.Y.)

**Keywords:** 8-hydroxy-2-anilino-1,4-naphthoquinone (8-HANQ), ferroptosis, cathepsin-B, neuroprotection, kainate (KA)-induced epileptic seizures, lipid ROS

## Abstract

**Background/Objectives:** Ferroptosis, an iron-dependent form of regulated cell death characterized by excessive lipid peroxidation, has been implicated in various acute and chronic brain disorders, including epilepsy. Although 1,4-naphthoquinone derivatives have been reported to regulate ferroptosis, their mechanistic roles in the nervous system remain underexplored. Here, we investigated the protective effects of 8-hydroxy-2-anilino-1,4-naphthoquinone (8-HANQ) on ferroptotic neuronal death in vitro and seizure behaviors in vivo. **Methods:** HT22 hippocampal cells were exposed to ferroptosis inducers including glutamate, glutamate plus iron, or RSL3. Lipid reactive oxygen species (ROS), ferroptosis markers, and its related molecules were assessed by flow cytometry and Western blotting. In a kainate (KA)-induced seizure model, 8-HANQ was delivered intracerebroventricularly, followed by behavioral seizure scoring and analysis of hippocampal levels of PSD95, cathepsin-B, and FGFR1 at 72 h post-seizure. **Results:** 8-HANQ attenuated ferroptotic death in HT22 cells, reducing lipid ROS accumulation and abnormal acyl-coA synthetase long chain family member 4 (ACSL4), suggesting 8-HANQ’s anti-ferroptotic action. Moreover, 8-HANQ also prevented aberrant STAT3-dependent cathepsin-B overexpression while modulating soluble N-cadherin-mediated FGFR1 activation. In vivo, 8-HANQ decreased KA-induced seizure behavior, restored hippocampal cathepsin-B and PSD95 expression, and partially alleviated dysregulation of FGFR1 activation. **Conclusions:** 8-HANQ prevents ferroptotic neuronal death and synaptic deficits involving FGFR1/STAT3/cathepsin-B-driven ferroptosis while lowering seizure severity, suggesting that 8-HANQ may serve as a potential anti-ferroptotic and anti-seizure agent.

## 1. Introduction

Naphthoquinones (NQs) are bioactive compounds structurally derived from naphthalene, and their numerous natural and synthetic derivatives have been extensively investigated for diverse biological effects and therapeutic potential. Among them, 1,4-NQs have attracted particular attention for their neuroprotective properties across various models of neurological disorders. Recently, a previous study has demonstrated that certain 1,4-NQ derivatives prevent Amyloid β (Aβ) aggregation, with their anti-Aβ activities being highly dependent on structural modifications [1]. In addition, structural analogues of 1,4-NQs have been shown to alleviate motor deficits and inhibit apoptosis reducing oxidative stress in rotenone-induced Parkinsonism models [2]. Moreover, synthetic vitamin K analogues, which are also 1,4-NQs, have been reported to reduce seizure activity in pentylenetetrazole-induced seizure models [3]. Extending their pharmacological scope to ferroptosis regulation, fully reduced forms of vitamin K have been identified as potent ferroptosis inhibitors by scavenging lipid radicals in a glutathione peroxidase 4 (GPX4)-independent manner in mouse hippocampal HT22 cells and brain tissues [4].

Ferroptosis is a distinct, non-apoptotic form of regulated cell death characterized by the excessive accumulation of intracellular iron and lipid reactive oxygen species (ROS). It has been implicated in various acute and chronic central nervous system (CNS) disorders [5]. During ferroptosis, iron overload amplifies lipid ROS generation, triggering extensive lipid peroxidation and cell death [6,7]. GPX4 plays a central antioxidant role by using glutathione (GSH) to detoxify lipid ROS, thereby protecting cells from ferroptotic damage [8]. In addition, acyl-CoA synthetase long-chain family member 4 (ACSL4) promotes the oxidation of polyunsaturated fatty acids (PUFAs), a key step in ferroptosis progression [9]. GPX4-independent pathways also contribute to ferroptosis regulation; for example, ferroptosis suppressor protein 1 (FSP1) uses coenzyme Q10 to neutralize lipid radicals [10,11].

Recent transcriptomic and functional studies have identified the lysosomal cysteine protease cathepsin-B as an executive mediator of ferroptosis in the CNS. Elevated expression and activity of cathepsin-B have been observed under ferroptotic conditions both in vitro and in vivo [12]. In HT22 neuronal cells, cathepsin-B promotes ferroptotic cell death by inducing lysosomal membrane permeabilization and enhancing lipid peroxidation, independently of GPX4 [13]. In non-neuronal systems, cathepsin-B expression is regulated by signal transducer and activator of transcription 3 (STAT3); the ferroptosis inducer erastin has been shown to cause lysosomal dysfunction and cathepsin-B upregulation via aberrant STAT3 activation in pancreatic cancer cells [14].

Additionally, N-cadherin, a classical cadherin and calcium-dependent adhesion molecule, has emerged as another modulator of ferroptosis. Under ferroptotic stress, N-cadherin degradation via autophagy reduces intercellular adhesion and increases susceptibility to cell death [15]. Ke et al. [16] also demonstrated that increased extracellular matrix stiffness decreases N-cadherin expression in nucleus pulposus cells, thereby enhancing lipid ROS, subsequent ferroptosis through upregulation of ACSL4.

To date, vitamin K analogues bearing long alkyl side chains have been the only naphthoquinone derivatives reported to regulate ferroptosis. 8-hydroxy-2-anilino-1,4-naphthoquinone (8-HANQ) is structurally distinct, characterized by an aniline substituent. Despite this distinctive structure, the biological effects of 8-HANQ, including its potential anti-ferroptotic and neuroprotective activities, remain largely unexplored. To elucidate its biological roles, the present study aims to define the neuroprotective role of 8-HANQ against ferroptosis in vitro and evaluate its potential anti-seizure effects in vivo.

## 2. Materials and Methods

### 2.1. Chemicals and Reagents

Glutamate, RAS-selective lethal 3 (RSL3), dimethyl sulfoxide (DMSO), Iron(II) sulfate heptahydrate (FeSO4), 3-(2-Pyridyl)-5,6-diphenyl-1,2,4-triazine-4′,4″-disulfonic acid sodium salt (Ferrozine), Iron(III) sulfate (iron (III)), deferoxamine (DFO), ethylenediaminetetraacetic acid (EDTA), ferrostatin-1, valproate and C^11^-BODIPY^581/591^ were purchased from Sigma-Aldrich (St. Louis, MO, USA). Antibodies against pSTAT3(Y705) (#9145), STAT3 (#9139), cathepsin-B (#31718), heme oxygenease-1 (HO-1; #82206), ferritin heavy chain-1 (FTH1; #4393), synapsin-1 (#5297), N-cadherin (#13116), fibroblast growth factor receptor 1 (FGFR1; #9740), microtubule-associated protein 1 light chain 3 B (LC3B; #3868) and β-actin (#3700) were purchased from Cell Signaling Technology (Danvers, MA, USA), pFGFR1(Y653/654) (#441140G) from Thermo Scientific (Waltham, MA, USA), postsynaptic density protein 95 (PSD95; #sc-32290) from SantaCruz (Dallas, TX, USA), FSP1 (#20886-1-AP) from Proteintech (Rosemont, IL, USA), GPX4 (#ab125066) from Abcam (Abcam, Cambridge, UK) and ACSL4 (#A6826), PSD95 (#A0131) for HT22 cells from ABclonal Technology (Woburn, MA, USA). BGJ398 and kainate (KA) was purchased from MedChemExpress (Monmouth Junction, NJ, USA).

### 2.2. NQ-Derived Compounds

All chemical structures of 6 compounds are shown in Supplementary Figure S1.

#### 2.2.1. 8-HANQ and 5-HANQ

##### General Chemistry

The reagents and solvents were purchased from commercial suppliers (Sigma-Aldrich, St. Louis, MO, USA; Thermo Fisher, Waltham, WA, USA; TCI, Tokyo, Japan) and used as provided, unless otherwise indicated. Reactions were monitored via analytical thin-layer chromatography (TLC) using glass sheets pre-coated with silica gel 60 F254 (Merck, Darmstadt, Germany) and visualized under ultraviolet (UV) light (254 nm). Proton nuclear magnetic resonance (^1^H NMR) spectra of the compounds dissolved in CDCl_3_ and deuterated dimethyl sulfoxide (DMSO-*d*_6_) were recorded on a Bruker Avance 400 MHz spectrometer (Bruker Corporation, Billerica, MA, USA). Chemical shifts were expressed as δ-values in parts per million (ppm) using residual solvent peaks (CDCl_3_: ^1^H, 7.26 ppm; DMSO: ^1^H, 2.50 ppm) as a reference. Coupling constants are given in hertz (Hz). Peak patterns are indicated by the following abbreviations: s, singlet; d, doublet; t, triplet; q, quadruplet; m, multiplet. HRMS was performed on a system consisting of an electrospray ionization (ESI) source in an Agilent 6230B time-of-flight (TOF) liquid chromatography–mass spectrometer equipped at the Ewha Drug Development Research Core Center (Agilent Technologies, Santa Clara, CA, USA; NFEC-2021-08-272459). Column chromatography was performed using silica gel 60 (230–400 mesh). The purity of the final compounds was determined by HPLC on an Agilent 1260 system (Agilent Technologies) using an Agilent Eclipse XDB-C18 column (4.6 × 150 mm, 5 µm) with UV absorbance detection at 254 nm. HPLC conditions were as follows: mobile phase, ACN/Water (60:40); flow rate, 1.0 mL/min; column temperature, ambient; injection volume, 10 μL. All compounds were >95% pure by HPLC analysis.

##### Synthesis

A solution of Juglone (200 mg, 1.1 mmol, 1 equiv.) and AlCl_3_(0.1 mmol, 0.1 equiv.) in ethanol (11 mL) was stirred at room temperature under dark conditions. Aniline (1.1 mmol, 1.0 equiv.) was then added, and the resulting mixture was stirred for 2 h. After the reaction was complete, the mixture was extracted with ethyl acetate and washed with brine. The organic phase was collected, dried over anhydrous MgSO_4_, filtered, and concentrated under reduced pressure. The crude product was purified by silica gel column chromatography using a gradient elution of hexane and ethyl acetate (30:1 to 20:1), yielding an orange-red compound (8-HANQ; 42 mg, 11% yield) and a dark red compound (5-Hydroxy-2-(phenylamino)naphthalene-1,4-dione, 5-HANQ; 5 mg, 2% yield) [17].

8-HANQ (compound #1): Orange red solid; ^1^H NMR (400 MHz, DMSO-*d*_6_) δ 11.53 (s, 1H), 9.27 (s, 1H), 7.74 (q, *J* = 5.3 Hz, 1H), 7.45 (t, *J* = 4.0 Hz, 3H), 7.38 (d, *J* = 4.3 Hz, 2H), 7.27–7.21 (m, 2H), 6.04 (s, 1H); ^1^H NMR (400 MHz, CDCl_3_) δ 11.56 (s, 1H), 7.65–7.62 (m, 2 H), 7.52 (s, 1H), 7.43 (t, *J* = 3.2 Hz, 2H), 7.27 (d, *J* = 7.7 Hz, 2H), 7.24–7.17 (m, 2H), 6.38 (s, 1H); The NMR data are identical to the previously reported data [17]; ^13^C NMR (100 MHz, DMSO-*d*_6_) δ 185.57, 181.89, 160.48, 146.27, 137.95, 137.55, 132.95, 129.32, 125.41, 123.89, 122.26, 117.60, 114.33, 102.14); HRMS (ES+) m/z calculated for C_16_H_12_NO_3_ [M+H]^+^: 266.0812; found: 266.0812. HPLC purity: 99.8%.

5-HANQ (compound #2): Dark red solid; ^1^H NMR (400 MHz, DMSO-*d_6_*) δ 13.06 (s, 1H), 9.60 (s, 1H), 7.66 (t, *J* = 7.8 Hz, 1H), 7.61 (d, *J* = 6.4 Hz, 1H), 7.48 (t, *J* = 7.8 Hz, 2H), 7.40 (d, *J* = 7.4 Hz, 2H), 7.33 (d, *J* = 7.2 Hz, 1H), 7.27 (t, *J* = 7.3 Hz, 1H), 6.00 (s, 1H); The NMR data are identical to the previously reported data [17,18]; ^13^C NMR (100 MHz, DMSO-*d*_6_) δ 189.05, 180.84, 160.00, 147.49, 137.62, 134.72, 130.57, 129.37, 125.84, 125.10, 124.06, 118.77, 114.27, 100.58; HRMS (ES+) m/z calculated for C_16_H_12_NO_3_ [M+H]^+^: 266.0812; found: 266.0811. HPLC purity: 96.4%.

#### 2.2.2. Other NQ-Derived Compounds

NQ-derived analogs, 6-anilino-5,8-quinolinedione (compound #3; [19]), 2,3-dimethyl-6-(3,5-difluoroanilino)-5,8-quinoxalinedione (compound #4), 2,3-dimethyl-6-(3-fluoroanilino)-5,8-quinoxalinedione (compound #5), and 2,3-dimethyl-6-(4-ethoxyanilino)-5,8-quinoxalinedione (compound #6) [20], were included in the initial compound screening and these 4 compounds were provided by Prof. Chung-Kyu Ryu.

### 2.3. HT22 Mouse Hippocampal Cell Culture

Cells were cultured in Dulbecco’s Modified Eagle’s medium containing 10% fetal bovine serum, 100 U/mL penicillin, and 100 mg/mL streptomycin. Cells were maintained at 37 °C in a humidified incubator under 5% CO_2_ and subcultured every 2–3 days using trypsin/EDTA solution.

### 2.4. Cell Viability Assay

Cell viability was assessed using the Quanti-Max™ WST-8 Cell Viability Assay Kit (Biomax, Guri-si, Republic of Korea), following the manufacturer’s protocol. HT22 cells were placed in 96-well plates and pre-treated with varying concentrations of each test compound including 8-HANQ (0.01–10 μM), 5-HANQ (10 μM), BGJ398 (1–5 nM) or ferrostatin-1 (1 μM) for 30 min and subsequently exposed to cytotoxic agents such as a combination of 5 mM glutamate and 100 μM iron (III), 50 nM RSL3 or 20 mM glutamate at 37 °C for 24 h. All compounds were dissolved in DMSO and diluted with phosphate-buffered saline (PBS, pH 7.4) before use, and the final DMSO concentration did not exceed 0.02% (*v*/*v*). To test toxicity of 8-HANQ and 5-HANQ, both compounds (10–100 μM) were treated to HT22 cells and incubated for 24 h. WST-8 reagent was added and incubated for 1 h in the dark and the absorbance at 450 nm was measured using a Tecan Infinite 200 PRO microplate reader (Tecan, Mannedorf, Switzerland; NFEC-2024-09-299675) at the Ewha Drug Development Research Core Center.

### 2.5. Lipid ROS Detection with C^11^-BODIPY^581/591^

HT22 cells were seeded in 6-well plates and pretreated with 8-HANQ or 5-HANQ (5–10 μM) for 30 min, followed by stimulation with either 30 mM glutamate or 200 nM RSL3 for 24 h. Cells were then fixed in 1% paraformaldehyde for 10 min at room temperature and stained with 1.5 μM C^11^-BODIPY^581/591^ at 37 °C for 30 min in the dark. After washing, cells were resuspended in PBS and analyzed by flow cytometry NovoCyte 2060R system (ACEA Biosciences, San Diego, CA, USA; NFEC-2019-03-254735). Fluorescence data from 100,000 events were recorded per sample. For fluorescence imaging, cells stained with 1.5 μM C^11^-BODIPY^581/591^ for 30 min were washed thoroughly with PBS and visualized using an Axio Observer 7 inverted fluorescence microscope (Carl Zeiss, Oberkochen, Germany; NFEC-2021-08-272462). All flow cytometric and fluorescence imaging analyses were conducted at the Ewha Drug Development Research Core Center.

### 2.6. Ferrous Iron Chelation Assay

Ferrous iron binding capacity of 8-HANQ was measured by the ferrozine-based colorimetric assay, as described by Soriano-Castell et al. [21]. Briefly, 8-HANQ (1–200 μM) was incubated with 5 μM FeSO4 in 50 mM HEPES buffer (pH 7.5) for 2 min at room temperature in a 96-well plate, followed by the addition of 2.5 mM ferrozine. Absorbance at 562 nm was measured using a microplate reader. Each dose was tested in duplicate and iron binding capacities are presented as vehicle controls with or without iron.

### 2.7. Measurement of Soluble N-Cadherin Release

Soluble N-cadherin was quantified as previously described by Nitsch et al. [22], with slight modifications. Briefly, cells were incubated in 6-well plates overnight, followed by treatment with 8-HANQ (2.5–10 μM) for 30 min prior to the addition of 200 nM RSL3. After 24 h, culture media were collected, centrifuged at 13,000× *g* for 10 min with 1% protease inhibitor cocktail and subsequently the supernatants were desalted using PD-10 desalting columns (Cytiva, Marlborough, MA, USA). The desalted solution from the columns was dried using a speed vacuum evaporator ScanSpeed 40 (Alex Red Ltd., Netanya, Israel) and resuspended in 20 μL of loading buffer. Equal volumes of each sample were separated with sodium dodecyl sulfate-polyacrylamide gel electrophoresis (SDS-PAGE), transferred to polyvinylidene difluoride (PVDF) membranes (Millipore, Burlington, MA, USA). Membranes were blocked with 5% non-fat dry milk in Tris-buffered saline with 0.1% Tween 20 (TBST) and probed with anti-N-cadherin primary antibody overnight. After incubation with horseradish peroxidase-conjugated secondary antibodies for 2 h, immunoactive bands were developed using enhanced chemiluminescence reagents and detected with a ChemiDoc MP imaging system (Bio-Rad, Hercules, CA, USA). Band intensities were analyzed using ImageJ software version 1.52a (NIH, Bethesda, MD, USA).

### 2.8. Animal Experiments

#### 2.8.1. Animals

Male ICR mice (25–30 g) were obtained from Orient Bio (Seongnam, Republic of Korea) and housed under a 12/12 h light/dark cycle with free access to standard chow and water. All animal procedures were approved by the Institutional Animal Care and Use Committee (IACUC) of Ewha Womans University (approval number: Ewha-IACUC 23-061-4) and conducted in accordance with institutional guidelines for the care and use of laboratory animals.

#### 2.8.2. Stereotaxic Drug Administration and KA-Induced Seizure Induction

For intracerebroventricular (i.c.v.) administration, mice were anesthetized with tiletamine-zolazepam (Zoletil, 20 mg/kg) and xylazine (9.5 mg/kg) and positioned in a stereotaxic frame. Injections were performed using a Hamilton microsyringe at the following stereotaxic coordinates relative to bregma: anteroposterior -1.0 mm, mediolateral ±1.0 mm, and dorsoventral −2.0 mm. Animals received a 1 μL injection of either 8-HANQ (0.5 or 1 μg), valproate (150 μg), or vehicle (40 or 80% DMSO in saline) with/without KA (0.2 μg).

Mice were intraperitoneally or intracerebroventricularly administered KA, as previously described by Rusina et al. [23]. In the intraperitoneal (i.p.) KA administration experiment, animals were randomly divided into four groups. The vehicle control group received an i.c.v. injection of vehicle 2 h prior to an i.p. injection of saline. The KA group received vehicle i.c.v., followed 2 h later by KA (40 mg/kg, i.p.). The KA + 8-HANQ group was administered 8-HANQ (0.5 or 1 μg, i.c.v.), and the KA + valproate group received valproate (150 μg, i.c.v.), both 2 h before KA injection.

In the i.c.v. KA administration experiment, a separate cohort of animals was randomly assigned into three groups. In this scheme, KA was co-administered with 8-HANQ via i.c.v. injection. The vehicle control group received an i.c.v. injection of vehicle (40% DMSO in 1 μL saline). The KA group received a mixture of KA (0.2 μg) and vehicle (in 1 μL total volume). The KA + 8-HANQ group was administered a combination of KA (0.2 μg) and 8-HANQ (1 μg) in a total volume of 1 μL. All animals were sacrificed 72 h after KA administration.

#### 2.8.3. Behavioral Seizure Assessment

To evaluate the anti-seizure effects of 8-HANQ in a KA-induced seizure model, mice were monitored every 10 min for 150 min by observers blinded to the treatment conditions. Behavioral seizure severity was scored between 30 and 180 min following systemic administration of KA. In the i.c.v. KA administration model, seizure behavior was evaluated from 60 to 210 min after i.c.v. injection of 8-HANQ and KA, as mice recovered from anesthesia approximately 60 min post-injection. Seizure severity was assessed using a modified Racine scale [24], as follows: Stage 0, no behavioral changes; Stage 1, facial muscle clonus; Stage 2, head nodding; Stage 3, forelimb clonus; Stage 4, rearing and falling with fore-limb clonus; Stage 5, generalized tonic–clonic seizures.

### 2.9. Sample Preparation and Western Blot Analysis

HT22 cells were seeded in 6-well plates and treated with 8-HANQ (2.5–20 μM) or BGJ398 (10–100 nM) for 30 min and then co-treated with 30 mM glutamate, 200 nM RSL3, or combinations of 10/20 mM glutamate or 100 μM arachidonate and 25/100 μM iron (III) for designated times. For in vivo analysis, mice were sacrificed 72 h after KA administration. Cells and brain hippocampal tissues were lysed in cold RIPA buffer containing 1% protease inhibitor cocktail. After centrifugation at 13,000× *g* for 20 min at 4 °C, protein concentrations were determined using a protein quantification kit—BCA (Biomax, Yongin, Republic of Korea). Equal protein amounts (20 μg) were separated by SDS-PAGE and transferred to PVDF membranes. Membranes were blocked with 5% non-fat dry milk in 0.1% TBST and incubated overnight with primary antibodies and visualized using enhanced chemiluminescence. Band intensity was quantified using ImageJ software.

### 2.10. Statistical Analysis

All data are expressed as mean ± standard error of the mean (SEM). The normality of data distribution was assessed using the Shapiro–Wilk test, and homogeneity of variances using the Brown–Forsythe test. Depending on the data distribution and experimental design, unpaired Student’s *t*-tests or one-way ANOVA followed by Tukey’s HSD test were applied. Planned pairwise comparisons were further conducted within a contrast analysis framework using Welch’s two-tailed *t*-tests to test predefined hypotheses. For each contrast, the *t*-statistic, degrees of freedom, exact *p*-value, and effect size (Cohen’s *d*) were calculated, with *p*-values adjusted using the Holm–Bonferroni method when multiple contrasts were planned. Statistical analyses were performed using GraphPad Prism 8.0 (GraphPad Software, Boston, MA, USA) and Microsoft Excel 2024 (Microsoft Corp., Redmond, WA, USA). A *p*-value < 0.05 was considered statistically significant.

### 2.11. Generative AI Assistance in Manuscript Preparation

Generative AI tools, specifically OpenAI’s ChatGPT 5 (San Francisco, CA, USA), were partially used to assist in the literature review and revision of the original manuscript. All AI-assisted content was carefully reviewed and edited by the authors to ensure accuracy.

## 3. Results

### 3.1. Selection of Novel NQ Derivatives Preventing Ferroptotic Neuronal Death

To identify novel NQ compounds inhibiting ferroptotic cell death, six synthetic NQ derivatives (compounds #1–-6) were tested for their neuroprotective effects against ferroptotic cell death in mouse hippocampal HT22 cells. The tested compounds included two 1,4-NQs, 8-HANQ (compound #1) and 5-HANQ (compound #2), a quinolinedione (compound #3), and three quinoxalinediones (compound #4–6).

**Figure 1:**
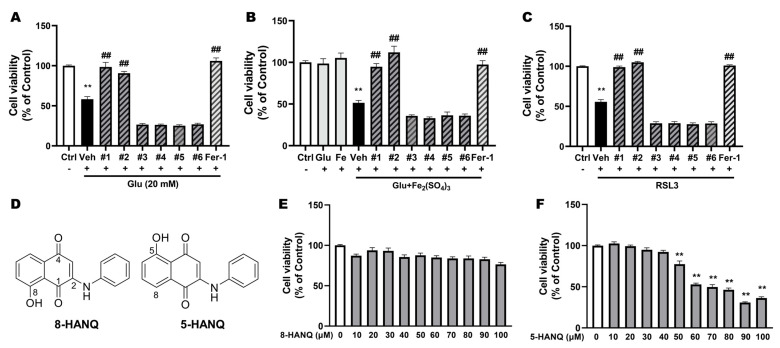
Selection of novel NQ derivatives preventing ferroptotic neuronal death (**A**–**C**) Cell viability was assessed using the WST-8 assay to identify candidate for ferroptosis inhibitors. HT22 cells were pretreated with 10 μM of various NQ derivatives (compound #1–6) or 1 μM ferrostatin-1 (Fer-1) for 30 min, followed by co-treatment with 20 mM glutamate (Glu; (**A**), n = 4), 5 mM Glu and 100 μM iron (III) sulfate (Fe_2_(SO_4_)_3_) ((**B**), n = 4) or 50 nM RSL3 ((**C**), n = 6) for 24 h. Fer-1 was used as positive control. (**D**) Chemical structures of 8-HANQ (compound #1) and 5-HANQ (compound #2). (**E**,**F**) Cytotoxicity of 8-HANQ ((**E**), n = 6) and 5-HANQ ((**F**), n = 4) was evaluated in the absence of ferroptosis inducers. HT22 cells were treated with increasing concentrations (10–100 μM) of each compound for 24 h, and viability was measured using the WST-8 assay. Statistical significance was determined by one-way ANOVA. Data are presented as means ± SEM. (** *p* < 0.01 vs. Control (Ctrl, 0); ## *p* < 0.01 vs. Glu, Glu + Fe_2_(SO_4_)_3_, RSL3). n indicates independent experiments.

Excessive glutamate is known to trigger multiple forms of cell death, including ferroptosis. At toxic concentration, glutamate induces ferroptosis by inhibiting system Xc−, thereby depleting GSH, and is therefore widely used as a ferroptosis inducer. Another hallmark of ferroptosis is the intracellular accumulation of iron [6]. To establish a more refined ferroptotic condition, we treated cells with a subtoxic concentration of glutamate in combination with iron. Among the six compounds, only #1 (8-HANQ) and #2 (5-HANQ) significantly prevented ferroptotic cell death induced either by glutamate alone (Figure 1A) or by glutamate plus iron (III) (Figure 1B). The two compounds also significantly attenuated neuronal death caused by RSL3, a selective GPX4 inhibitor and established ferroptosis inducer (Figure 1C). Treatment with 8-HANQ or 5-HANQ at 10 μM almost fully restored neuronal viability to the level achieved by ferrostatin-1, a well-characterized anti-ferroptotic agent [6].

8-HANQ and 5-HANQ are structural isomers that differ in the position of the aniline group on the 1,4-NQ scaffold (Figure 1D). Both compounds were synthesized as described in the Materials and Methods, and their intrinsic cytotoxicity was assessed under normal conditions. When HT22 cells were treated with concentrations ranging from 10 to 100 μM, 8-HANQ showed no detectable toxicity (Figure 1E), whereas 5-HANQ caused dose-dependent toxicity beginning at 50 μM (Figure 1F). Therefore, 8-HANQ inhibits ferroptotic neuronal death with a more favorable toxicity profile than 5-HANQ, and thus was selected for further mechanistic studies.

### 3.2. 8-HANQ Exhibits Dose-Dependent Neuroprotection Against Ferroptotic Neuronal Death and Synaptic Dysregulation in HT22 Cells

**Figure 2:**
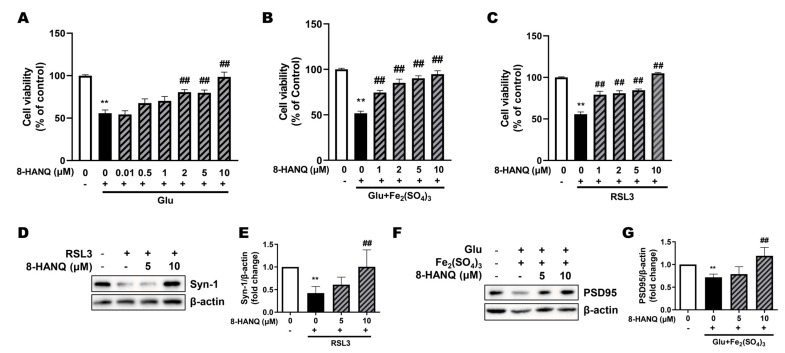
Protection against ferroptosis and preservation of synaptic proteins by 8-HANQ (**A**–**C**) Dose-dependent neuroprotective effects of 8-HANQ were assessed in toxic concentration of glutamate- (Glu; (**A**), n = 5), subtoxic combination of Glu and iron (III) sulfate (Fe_2_(SO_4_)_3_)- ((**B**), n = 6), and RSL3- ((**C**), n = 4) induced ferroptosis models. 8-HANQ (0.01–10 μM) was applied 30 min prior to treatment with 20 mM Glu, 5 mM Glu and 100 μM Fe_2_(SO_4_)_3_ or 50 nM RSL3 and incubated for 24 h. (**D**–**G**) Western blot analysis and quantification of synaptic markers including synapsin-1 (Syn-1; D-E, n = 4) and PSD95 ((**F**,**G**), n = 6) expression. Cells were pretreated with 8-HANQ (5–10 μM) or for 30 min following co-treatment with 200 nM RSL3 or 20 mM Glu and 25 μM Fe_2_(SO_4_)_3_ for 24 h. Data are presented as means ± SEM. Statistical significance was assessed using one-way ANOVA (**A**–**C**) or planned pairwise contrasts (Welch’s *t*-tests with Holm–Bonferroni correction; (**D**–**G**)). (** *p* < 0.01 vs. Control (0); ## *p* < 0.01 vs. Glu, RSL3, Glu + Fe_2_(SO_4_)_3_). n indicates independent experiments.

We confirmed the anti-ferroptotic neuroprotective effects of 8-HANQ across different concentrations in HT22 cells. 8-HANQ protected against glutamate-induced ferroptosis in a dose-dependent manner, showing significant effects even at a low concentration of 2 μM (Figure 2A). Comparable protective effects were also observed under ferroptotic conditions induced either by the combination of subtoxic glutamate with iron (III) (Figure 2B) or by RSL3 (Figure 2C), with significant effect beginning at 1–2 μM.

We next examined whether 8-HANQ could preserve synapse marker expression, namely presynaptic synapsin-1 and postsynaptic PSD95, which reflect synaptic strength [25], during ferroptosis. Consistent with its protective effects, 8-HANQ restored the levels of both proteins (Figure 2D–G), which had been reduced by glutamate plus iron (III) or RSL3. These findings suggest that 8-HANQ provides neuroprotection while maintaining synaptic strength under ferroptotic stress.

### 3.3. 8-HANQ Suppresses Lipid ROS Accumulation to Inhibit Ferroptosis

**Figure 3:**
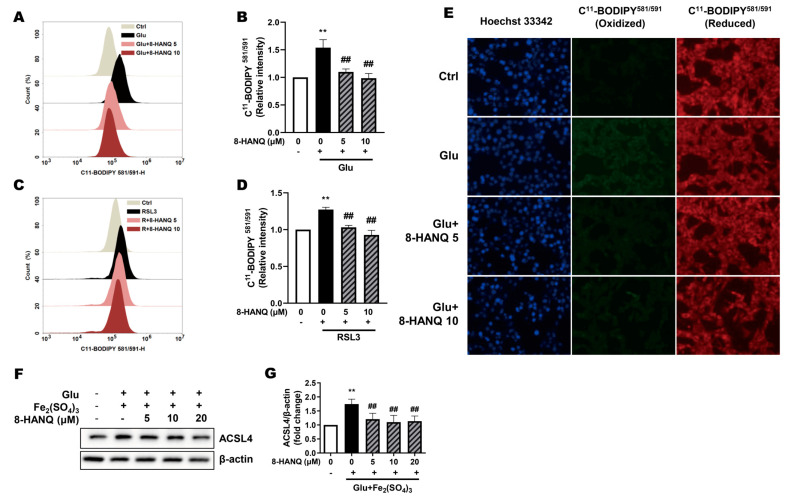
Suppression of increased ferroptosis-associated lipid ROS and ACSL4 levels by 8-HANQ. (**A**–**D**) Lipid ROS levels were measured using C^11^-BODIPY^581/591^ fluorescence with flow cytometry. 8-HANQ (5–10 μM) was applied 30 min prior to treatment with 30 mM glutamate (Glu; (**A**,**B**), n = 7) or 200 nM RSL3 ((**C**,**D**), n = 8) for 24 h. Statistical significance was determined by one-way ANOVA. (**E**) Representative fluorescence images of C^11^-BODIPY^581/591^ staining obtained after 24 h of Glu (30 mM) exposure with or without 8-HANQ pretreatment (5–10 μM). Scale bar: 50 μm (n = 4). (**F**,**G**) Western blot analysis and quantification of ACSL4. HT22 cells were pretreated with 8-HANQ (5–20 μM) 30 min, followed by exposure to 20 mM Glu and 25 μM iron (III) sulfate (Fe_2_(SO_4_)_3_) for 24 h (n = 4). Data are presented as means ± SEM. Statistical significance was assessed using planned pairwise comparisons (Welch’s two-tailed *t*-tests with Holm–Bonferroni correction). (** *p* < 0.01 vs. Control (Ctrl, 0); ## *p* < 0.01 vs. Glu, RSL3, Glu + Fe_2_(SO_4_)_3_). n indicates independent experiments.

Given the involvement of lipid ROS in ferroptosis, we assessed the ability of 8-HANQ to suppress ROS accumulation in glutamate- or RSL3-treated HT22 cells. Indeed, 8-HANQ significantly reduced lipid ROS levels under both conditions, as measured by flow cytometry using C^11^-BODIPY^581/591^ (Figure 3A–D). This lipid ROS-lowering effect of 8-HANQ was further confirmed in glutamate-treated cells by fluorescence microscopy analysis (Figure 3E).

ACSL4 promotes the oxidation of PUFAs, a critical step in ferroptosis progression [9]. To assess whether the reduction in lipid ROS was linked to alterations in lipid metabolism, we evaluated ACSL4 expression. The increase in ACSL4 induced by the combination of glutamate with iron (III) was markedly suppressed by 8-HANQ (Figure 3F,G). These findings suggest that 8-HANQ may exert anti-ferroptotic effects by modulating lipid metabolism through the inhibition of aberrant ACSL4-mediated lipid peroxidation.

### 3.4. 8-HANQ Inhibits the STAT3/Cathepsin-B Axis Mediated by Soluble N-Cadherin/FGFR1 Signaling to Attenuate Ferroptosis

**Figure 4:**
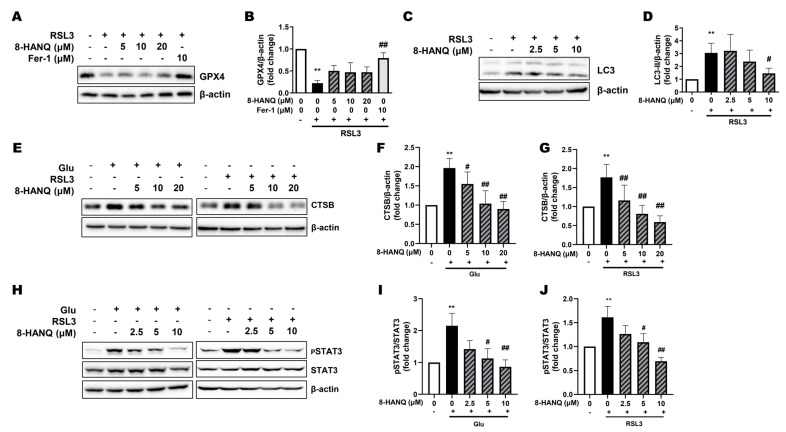
Modulation of STAT3-dependent cathepsin-B overexpression by 8-HANQ independently of GPX4 regulation. (**A**–**D**) Western blot analysis of GPX4 ((**A**,**B**), n = 3) and LC3-II ((**C**,**D**), n = 5) expression in RSL3-induced ferroptotic HT22 cells. Cells were pretreated with 8-HANQ (2.5–20 μM) or 10 μM ferrostatin-1 (Fer-1) for 30 min, followed by co-treatment with 200 nM RSL3 for 24 h. (**E**–**J**) Western blot images and quantification of cathepsin-B (CTSB) levels ((**E**–**G**); (**F**), n = 4; (**G**), n = 5) and pSTAT3/STAT3 ((**H**–**J**); (**I**), n = 5; (**J**), n = 6). Cells were pretreated with 8-HANQ (2.5–20 μM) for 30 min, followed by co-incubation with either 30 mM glutamate (Glu) or 200 nM RSL3 for 24 h. Data represent means ± SEM. Statistical significance was assessed using planned pairwise comparisons (Welch’s two-tailed *t*-tests with Holm–Bonferroni correction; (**A**–**G**)) or one-way ANOVA (**H**–**J**). (** *p* < 0.01 vs. Control (0); # *p* < 0.05 and ## *p* < 0.01 vs. Glu, RSL3). n indicates independent experiments.

To further delineate the mechanism underlying 8-HANQ’s anti-ferroptotic action, we first examined the involvement of GPX4. 8-HANQ did not restore the RSL3-induced reduction in GPX4 protein levels, which is associated with diminished enzymatic activity [8], whereas ferrostatin-1 effectively rescued GPX4 expression (Figure 4A,B). These results indicate that the anti-ferroptotic effect of 8-HANQ is not mediated through direct regulation of GPX4.

Since STAT3-mediated regulation of cathepsin-B has been implicated in ferroptosis of cancer cells [14], we next investigated their roles in HT22 neuronal ferroptosis and the action of 8-HANQ. Previous studies reported that cathepsin-B contributes to autophagy-dependent ferroptosis in HT22 cells [13]. Consistent with this, we found that LC3-II accumulation induced by RSL3 was markedly reduced by 8-HANQ (Figure 4C,D). Likewise, the abnormal increase in cathepsin-B expression caused by glutamate or RSL3 was significantly attenuated by 8-HANQ (Figure 4E–G). STAT3 activation paralleled cathepsin-B upregulation in ferroptotic conditions, and both were suppressed by 8-HANQ (Figure 4H–J). Together, these findings suggest that 8-HANQ exerts its anti-ferroptotic effect, at least in part, by inhibiting the STAT3/cathepsin-B axis, independently of GPX4 modulation.

**Figure 5:**
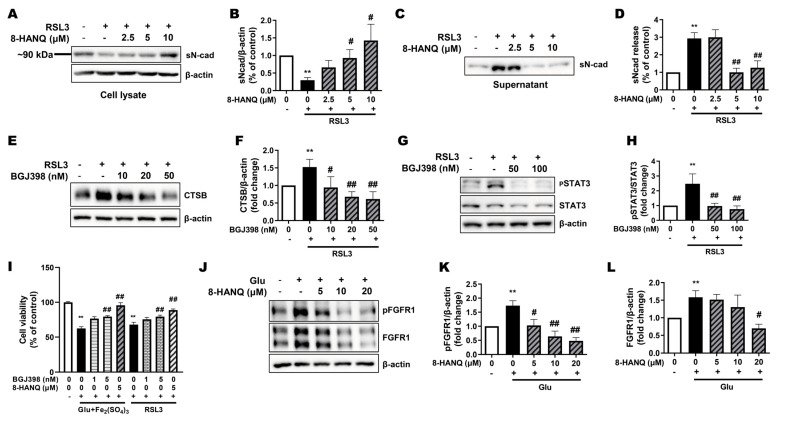
Suppressive effect of 8-HANQ on increased levels of FGFR1, pSTAT3/STAT3, cathepsin-B and soluble N-cadherin release during ferroptosis. (**A**–**D**) Western blot analysis and quantification of soluble N-cadherin in cell lysates ((**A**,**B**), n = 7) and culture supernatants ((**C**,**D**), n = 7). Cells were pretreated with 8-HANQ (2.5–10 μM) for 30 min, followed by exposure to 200 nM RSL3 for 24 h. Supernatants were concentrated from cell culture media using desalting columns prior to analysis. (**E**–**H**) Western blot analysis and quantification of CTSB expression ((**E**,**F**), n = 3) and pSTAT3/STAT3 ((**G**,**H**), n = 4) following BGJ398 treatment during ferroptosis. Cells were pretreated with BGJ398 (10–100 nM) for 30 min before exposure to 200 nM RSL3 for 4 h (pSTAT3/STAT3) or 24 h (CTSB). (**I**) Measurement of cell protection of BGJ398 using the WST-8 assay. BGJ398 (1–5 nM) or 8-HANQ (5 μM) was added 30 min before exposure to 5 mM glutamate (Glu) and 100 μM iron (III) sulfate (Fe_2_(SO_4_)_3_) or 50 nM RSL3 (n = 6). (**J**–**L**) Western blot analysis and quantification of pFGFR1 and total FGFR1 following 8-HANQ (5–20 μM) treatment for 2 h and 30 mM Glu exposure for 6 h (n = 4). Data represent means ± SEM. Statistical significance was assessed using one-way ANOVA (**A**–**D**,**I**,**J**–**L**) or planned pairwise comparisons (Welch’s two-tailed *t*-tests with Holm–Bonferroni correction; (**E**–**H**)). (** *p* < 0.01 vs. Control (0); # *p* < 0.05 and ## *p* < 0.01 vs. RSL3, Glu + Fe_2_(SO_4_)_3_, Glu). n indicates independent experiments.

Meanwhile, N-cadherin has recently been identified as a mechanosensitive regulator of ferroptosis [15,16]. We therefore assessed its expression in HT22 cells under ferroptotic stress. Although the full-length 140 kDa form remained unchanged following RSL3 treatment (Supplementary Figure S2A,B), the ~90 kDa soluble N-cadherin fragment in the cell pellet was consistently reduced, and this effect was reversed by 8-HANQ (Figure 5A,B). Because soluble N-cadherin is released extracellularly [26], this reduction likely reflected increased secretion, which was confirmed by elevated extracellular levels in RSL3-treated cells and restored to baseline by 8-HANQ (Figure 5C,D).

FGFR1, a known receptor for soluble N-cadherin, promotes neurite outgrowth through FGFR1-dependent signaling [27] and has been shown to activate STAT3 signaling pathways that govern cell fate decisions [28,29]. We hypothesized that FGFR1 serves as a mechanistic link between soluble N-cadherin release and activation of the STAT3/cathepsin-B axis during ferroptosis. Supporting this, pharmacological inhibition of FGFR1 with BGJ398 [28] suppressed RSL3-induced STAT3 and cathepsin-B upregulation in HT22 cells (Figure 5E–H). Moreover, BGJ398 protected neurons from ferroptosis triggered by either glutamate plus iron (III) or by RSL3 (Figure 5I). Both total and phosphorylated FGFR1 levels were elevated under glutamate-induced ferroptotic stress, and this upregulation was attenuated by 8-HANQ (Figure 5J–L). Collectively, these findings suggest that soluble N-cadherin-mediated activation of FGFR1 contributes to neuronal ferroptosis via the STAT3/cathepsin-B axis and that 8-HANQ protects neurons by disrupting this signaling cascade.

### 3.5. 8-HANQ Mitigates Seizure Severity and Restores Hippocampal Cathepsin-B, FGFR1, and PSD95 Dysregulation in KA-Induced Epileptic Mice

Recent studies have increasingly implicated ferroptosis in the pathogenesis of epilepsy [30]. In KA-induced seizure models, alterations in ferroptosis-related proteins such as GPX4 and ACSL4 have been reported [31,32,33]. These ferroptosis-associated alterations are closely linked to neuronal degeneration, and their reversal by genetic or pharmacological modulation has been shown to alleviate seizure severity, supporting the role of ferroptosis inhibition in anti-seizure activity. In addition, hippocampal cathepsin-B expression is elevated after KA-induced seizures, and its pharmacological inhibition has been shown to alleviate seizure-associated neuronal injury [34].

**Figure 6:**
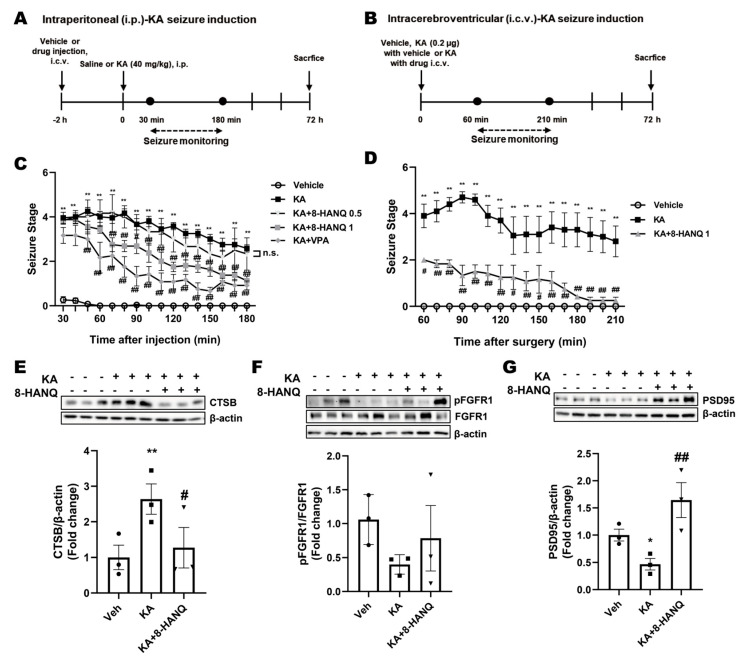
Anti-seizure and neuroprotective effects of 8-HANQ in KA-induced mice. (**A**) Schematic illustration of the experimental design for assessing the protective effect of 8-HANQ in intraperitoneal (i.p.) kainate (KA)-induced seizure model. (**B**) Schematic illustration of the experimental design for evaluating the protective effect of 8-HANQ in intracerebroventricular (i.c.v.) injection of KA-induced seizure model. (**C**) Mice received i.c.v. injections of vehicle (Veh; 80% DMSO in saline, 1 μL; n = 13), 8-HANQ (0.5 μg, n = 3; 1 μg, n = 8), or valproate (VPA; 150 μg, n = 6) 2 h prior to i.p. administration of KA (40 mg/kg; n = 12). Seizure behavior was monitored every 10 min for 150 min (from 30 min to 180 min after KA injection). Valproate was used as a positive control. (**D**) Mice were administered Veh (40% DMSO in saline, 1 μL; n = 3) or 8-HANQ (1 μg, n = 3) together with 0.2 μg KA (n = 4) via i.c.v. injection. Seizure scores were recorded every 10 min for 150 min (from 60 to 210 min after surgery). Data are presented as mean ± SEM. Statistical significance was determined. (**E**–**G**) Western blot analysis and quantification of cathepsin-B (CTSB; (**E**)), pFGFR1/FGFR1 ratio (**F**), and PSD95 (**G**) in hippocampal tissues collected 72 h after intracerebroventricular KA administration. Data are presented as mean ± SEM from 3 mice per group. Statistical significance was assessed using planned pairwise comparisons (Welch’s two-tailed *t*-tests with Holm–Bonferroni correction). (* *p* < 0.05, and ** *p* < 0.01 vs. Veh; # *p* < 0.05, and ## *p* < 0.01 vs. KA).

To investigate whether 8-HANQ exerts protective effects against KA-induced seizures, we employed two experimental models in which KA was administered either intraperitoneally or intracerebroventricularly, following methods similar to those described by Rusina et al. [23]. The effect of intracerebroventricularly delivered 8-HANQ was evaluated by behavioral seizure scoring, with valproate, a broad-spectrum anticonvulsant, included as a reference control. In the systemic KA model, mice received 8-HANQ or valproate two hours before intraperitoneal KA injection, and seizure behaviors were subsequently monitored (Figure 6A). The antiseizure effect of 8-HANQ was further confirmed in the intracerebroventricular KA model, where 8-HANQ (1 µg) was co-administered with KA (Figure 6B).

Systemic KA injection induced robust seizures (scores 4.3–2.6), which were significantly reduced by pretreatment with 8-HANQ (1 µg; scores 2.8–1.1) during the 70–150 min post-induction period, comparable to the reduction observed in valproate-treated mice (scores 2.3–0.9; Figure 6C). Similarly, intracerebroventricular KA injection produced seizure severity scores (4.7–2.8) consistent with systemic administration, and co-administration of 8-HANQ significantly attenuated seizure severity (scores 2.4–0.6) throughout the 60–210 min monitoring window (Figure 6D).

Given our in vitro findings that 8-HANQ normalizes ferroptosis-associated changes in ACSL4, cathepsin-B, and FGFR1, we next examined whether these effects were reproduced in hippocampal tissue from KA-induced epileptic mice. Under our experimental conditions involving intracerebroventricular KA administration with anesthesia, we did not detect alterations in GPX4 or ACSL4, limiting assessment of 8-HANQ effects on these proteins (Supplementary Figure S3A,B). However, 8-HANQ significantly suppressed KA-induced hippocampal cathepsin-B upregulation (Figure 6E) and partially attenuated the trend toward a decreased pFGFR1/FGFR1 ratio (Figure 6F). Moreover, 8-HANQ preserved synaptic proteins, as evidenced by enhanced expression of the postsynaptic marker PSD95, which was reduced by intracerebroventricular injection of KA in vivo (Figure 6G). These results are consistent with the protective actions of 8-HANQ observed under in vitro ferroptotic stress conditions. Taken together, these findings indicate that 8-HANQ not only reduces seizure severity but also counteracts hippocampal dysregulation of cathepsin-B, FGFR1, and PSD95, which may underlie its antiseizure effects in vivo.

## 4. Discussion

Due to their structural versatility, 1,4-NQ derivatives exert diverse biological effects on ferroptosis in both cancer and neuronal systems [1,4,35,36]. While some 1,4-NQs such as juglone and plumbagin promote ferroptosis by inducing GPX4 degradation [35,36], they have been reported to exert anti-oxidative neuroprotective effects in oxygen-glucose deprivation model at submicromolar concentrations (0.1–1 μM) but become cytotoxic at higher doses [37,38]. To date, vitamin K analogs remain the only 1,4-NQ derivatives known to exhibit anti-ferroptotic activity, acting through the FSP1-dependent pathway [4]. 8-HANQ exhibited neuroprotective, anti-ferroptotic activity without restoring GPX4 or FSP1 levels, implying a distinct mechanism from previously known analogues. However, the role of 8-HANQ in ferroptosis has not been investigated, and no notable function has been described in the neuronal system. To date, only one prior study reported a negligible anti-Aβ aggregation effect of 8-HANQ in a cell-free assay [17], without subsequent evaluation in disease-relevant models. To our knowledge, no other reports have described any biological activity or neuroprotective efficacy of 8-HANQ, indicating that the present study provides the first experimental evidence of its ferroptosis-related neuroprotective action.

In the present study, we demonstrated that 8-HANQ attenuates neuronal ferroptosis in vitro, potentially by suppressing aberrant STAT3/cathepsin-B activation downstream of soluble N-cadherin/FGFR1 signaling in hippocampal neurons. Furthermore, in a KA-induced seizure model, 8-HANQ reduced seizure severity in vivo, underscoring the translational relevance of this mechanism.

We first evaluated the anti-ferroptotic effects of 8-HANQ under ferroptotic conditions induced by glutamate alone, glutamate combined with iron, and GPX4 inhibition by RSL3. Given the multifactorial nature of ferroptosis [39], we newly established a combined subtoxic glutamate and iron model, which synergistically elicited ferroptotic stress by simultaneously inhibiting system Xc^−^ and promoting iron-dependent lipid peroxidation. 8-HANQ dose-dependently prevented HT22 neuronal death across all conditions, in association with reduced lipid ROS accumulation. Moreover, 8-HANQ markedly suppressed aberrant ACSL4 upregulation, which facilitates PUFA oxidation and subsequent lipid peroxidation [9].

Despite these robust protective effects, 8-HANQ did not restore GPX4 protein levels under RSL3-induced stress. Because vitamin K derivatives, which also share a 1,4-NQ core, suppress ferroptosis via the GPX4-independent FSP1 pathway in HT22 cells [4], we next examined FSP1 involvement. However, 8-HANQ failed to restore FSP1 levels suppressed by arachidonate plus iron (III), another ferroptosis-inducing condition [40]. In addition, HO-1, whose overexpression aggravates ferroptosis through iron dysregulation [39], remained unaffected by 8-HANQ, and the compound exhibited no iron-chelating activity (Supplementary Figure S4). Together, these findings suggest that 8-HANQ exerts its protective effects through mechanisms distinct from canonical ferroptosis regulators such as GPX4, FSP1, or iron-handling pathways.

Instead, 8-HANQ suppressed pSTAT3 and cathepsin-B overactivation under both glutamate- and RSL3-induced ferroptosis. cathepsin-B upregulation has been consistently observed during ferroptosis across diverse cell types [12,13,41]. Concomitant induction of pSTAT3 and cathepsin-B has also been reported in pancreatic cancer cells undergoing ferroptosis [14], consistent with our findings in neuronal HT22 cells. Although STAT3 signaling in ferroptosis is context-dependent [42], growing evidence suggests a pro-ferroptotic role of pSTAT3 in CNS disorders. For example, activation of the STAT3/HIF1α axis promoted α-synuclein-induced ferroptosis, with GPX4 suppression and iron dyshomeostasis in a Parkinson’s disease model, whereas pharmacological STAT3 inhibition reduced lipid ROS and iron accumulation in microglia [43]. Likewise, traumatic brain injury-induced IL-23 upregulation enhanced neuronal ferroptosis through STAT3 activation, while IL-23 neutralization attenuated ferroptosis, restored GPX4 and ACSL4 expression, and normalized STAT3 overactivation [44]. Compared with these reports, our results indicate that 8-HANQ normalizes STAT3/cathepsin-B overactivation while restoring ACSL4 and reducing lipid ROS, but without altering GPX4 levels or iron homeostasis in ferroptosis-induced HT22 neuronal cells.

The autophagic degradation of N-cadherin has recently been identified in cancer cell lines as a mechanism of ferroptosis, mediated by the selective cargo protein hippocalcin-like 1 (HPCAL1) and operating independently of GPX4 regulation or iron metabolism [15]. Given that 8-HANQ attenuated LC3-II accumulation under ferroptotic stress, its anti-ferroptotic actions may be associated with suppression of autophagy-dependent ferroptosis, which led us to investigate N-cadherin protein level. However, intracellular levels of full-length N-cadherin and HPCAL1 remained unchanged (Supplementary Figure S2), despite decreased GPX4 level and dysregulated iron metabolism (Figure 4 and Supplementary Figure S4) under our ferroptotic conditions. These results indicated that HPCAL1-mediated N-cadherin degradation is not involved in neuronal ferroptosis. Instead, we observed a robust secretion of soluble N-cadherin, which has been reported to activate FGFR1 signaling and promote neurite outgrowth in cerebellar neurons [27]. Consistent with this interaction, our data demonstrated FGFR1 activation concomitant with elevated soluble N-cadherin under ferroptotic stress, an effect that was significantly inhibited by 8-HANQ. Based on these findings, we hypothesized that soluble N-cadherin may act as an upstream modulator of FGFR1/STAT3/cathepsin-B axis, thereby driving pathological STAT3/cathepsin-B activation during neuronal ferroptosis. Likewise, although arising from distinct cellular contexts, aberrant FGFR1/STAT3 activation has been shown to trigger cell death in breast cancer cells [45]. Importantly, our data suggest that 8-HANQ may attenuate ferroptosis-induced soluble N-cadherin secretion and the subsequent STAT3/cathepsin-B activation, together with its anti-ferroptotic neuroprotective effect. This pattern was comparable to the effects observed with BGJ398, an FGFR1 inhibitor. Collectively, our findings suggest that 8-HANQ may exert anti-ferroptotic and neuroprotective effects, at least in part, by attenuating soluble N-cadherin–mediated FGFR1 activation and the subsequent STAT3/cathepsin-B overexpression under ferroptotic conditions in vitro. Nevertheless, it remains possible that these effects occur secondarily to the modulation of redox homeostasis rather than direct inhibition of the FGFR1/STAT3/cathepsin-B axis.

Previous reports have demonstrated anti-seizure effect of certain 1,4-NQ derivatives in pentylenetetrazole-induced [3] and electroshock-induced seizures [46]. In addition, although there have been no reports in epilepsy or seizure models, the 1,4-NQ derivative, plumbagin, was recently shown to exert its neuroprotective effects in the hippocampus of autism models, ameliorating cognitive dysfunction [47].

To date, the potential effects of 1,4-NQ derivatives on seizure behavior in the context of neuroprotection against ferroptosis have not been investigated in vivo seizure models. In our in vitro neuronal ferroptosis model, synaptic dysfunction, as indicated by PSD95 reduction, was recovered by 8-HANQ. Consistent with this observation, 8-HANQ attenuated hippocampal PSD95 loss in parallel with its antiseizure effect in KA-induced epileptic mice. PSD95 has been reported to decline progressively as epilepsy advances. Previous studies indicated that no significant changes were detected 24 h after KA exposure [48], whereas a marked reduction was observed at one week in electrically induced seizures [49] and at six weeks after KA injection [50], indicating chronic synaptic impairment in epilepsy. In line with these reports, we observed PSD95 reduction at 72 h after KA injection, which was mitigated by 8-HANQ treatment.

In the KA models, 8-HANQ also attenuated seizure severity while modulating cathepsin-B levels, suggesting a potential relationship with ferroptotic processes. HO-1, a ferroptosis marker protein, was upregulated in hippocampal tissues from both intraperitoneal and intracerebroventricular KA-induced epileptic mice, which were anesthetized prior to vehicle or drug injection. In contrast, significant changes in GPX4 and ACSL4 were not detected in the same epileptic mice (unpublished data and Supplementary Figure S3). This absence of alteration suggests incomplete ferroptotic activation, possibly due to anesthetic interference, as ketamine, mechanistically related to tiletamine, has been reported to suppress neuronal ferroptosis [51]. Nonetheless, 8-HANQ reduced epileptic behavior while attenuating KA-induced cathepsin-B upregulation in the hippocampus, consistent with its anti-ferroptotic actions observed in vitro. Previous studies have shown that cathepsin-B is elevated following KA-induced seizures [34] and that it promotes ferroptosis in HT22 cells, where its inhibition alleviates ferroptotic death independently of GPX4 [13]. By integrating these observations, the present study provides an evidence for a potential link between cathepsin-B and ferroptosis in the context of epileptic seizures.

Previously, it has been demonstrated that KA-induced seizures activate STAT3 in association with glutamate excitotoxicity and neuroinflammation three days post-injection and that a traditional herbal formula restored these pathological changes [52]. Based on these findings, we assessed STAT3 signaling in the KA-exposed hippocampus. However, significant alterations in pSTAT3 were not observed in the KA-induced hippocampus (Supplementary Figure S3), limiting interpretation of its contribution to the in vivo effects of 8-HANQ.

In parallel, hippocampal FGFR1 signaling was also affected by seizure induction. At 72 h after KA injection, pFGFR1/FGFR1 ratios slightly declined, although not significantly, and were partially restored by 8-HANQ. These findings contrast with our in vitro data and earlier reports showing FGFR1 upregulation within 24 h of KA-induced seizures [53,54]. To date, FGFR1 signaling has not yet been examined in chronic epilepsy. Our results suggest that FGFR1 signaling downregulation may be indicative of progressive synaptic deterioration, whereas partial recovery by 8-HANQ treatment may contribute to neuroprotection.

In conclusion, our findings suggest that 8-HANQ mitigates neuronal ferroptosis, likely involving the FGFR1/STAT3/cathepsin-B axis rather than the canonical GPX4 or FSP1 defense systems. The reduction in lipid ROS and aberrant FGFR1/STAT3/cathepsin-B activation in vitro, together with the alleviation of synaptic deficits and seizure behaviors in vivo, provides preliminary support for a previously underexplored regulatory mechanism of ferroptosis associated with 8-HANQ. Taken together, these results raise the possibility that 8-HANQ may help control ferroptosis-related neuronal death and seizures, and further optimization of its derivatives to improve bioavailability and pharmacokinetic properties, along with in vivo toxicity evaluation, will be essential to assess future translational potential.

## Data Availability

The datasets generated during the current study are available from the corresponding authors upon request.

## Supplementary Materials

The following supporting information can be downloaded at: https://www.mdpi.com/article/10.3390/pharmaceutics17111415/s1, Figure S1: Structures of tested NQ-derived compounds, Figure S2: Unchanged expression of full-length N-cadherin and hippocalcin-like 1 during neuronal ferroptosis, Figure S3: Unaffected expression of GPX4, ACSL4, and pSTAT3, with induction of HO-1 after KA administration, Figure S4: Lack of involvement of FSP1, iron metabolism, and iron chelation in the neuroprotective anti-ferroptotic action of 8-HANQ.

## Abbreviations

The following abbreviations are used in this manuscript:

5-HANQ	5-Hydroxy-2-anilino-1,4-naphthoquinone
8-HANQ	8-Hydroxy-2-anilino-1,4-naphthoquinone
ACSL4	Acyl-CoA Synthetase Long-chain Family Member 4
Aβ	Amyloid β
CNS	Central Nervous System
DFO	Deferoxamine
EDTA	Ethylenediaminetetraacetic Acid
FGFR1	Fibroblast Growth Factor Receptor 1
FSP1	Ferroptosis Suppressor Protein 1
FTH1	Ferritin Heavy Chain 1
GPX4	Glutathione Peroxidase 4
GSH	Glutathione
HO-1	Heme Oxygenase-1
HPCAL1	Hippocalcin-like 1
iron (III)	Iron(III) Sulfate
KA	Kainic Acid
LC3	Microtubule-associated protein 1 light chain 3
NQ	Naphthoquinone
PSD95	Postsynaptic Density Protein 95
PUFA	Polyunsaturated Fatty Acid
ROS	Reactive Oxygen Species
RSL3	RAS-selective lethal 3
STAT3	Signal Transducer and Activator of Transcription 3
