# Supplementary material for: 8-Hydroxy-2-Anilino-1,4-Naphthoquinone Prevents Against Ferroptotic Neuronal Death and Kainate-Induced Epileptic Seizures
Source: Pharmaceutics. 2025 Oct 31;17(11):1415. doi: 10.3390/pharmaceutics17111415 (PMC12655069; doi:10.3390/pharmaceutics17111415)
Supplement: Supplementary file 1 [file pharmaceutics-17-01415-s001.zip › pharmaceutics-3929913-supplementary.pdf]

pharmaceutics

Article

# 8-Hydroxy-2-Anilino-1,4-Naphthoquinone Prevents Against Ferroptotic Neuronal Death and Kainate-Induced Epileptic Seizures

**Daseul Lee, Eun Jung Na, Yumi Heo, Jinha Yu and Hwa-Jung Kim ***

College of Pharmacy and Graduate School of Pharmaceutical Sciences, Ewha Womans University, Seoul 03760, Republic of Korea; dsleee@ewha.ac.kr (D.L.); ejna@wha.ac.kr (E.J.N.); ymheo0710@ewhain.net (Y.H.); jhyu@ewha.ac.kr (J.Y.)

* Correspondence: hjkim@ewha.ac.kr (H-J.K); Tel.: +82-2-3277-3021

## Supplementary Materials

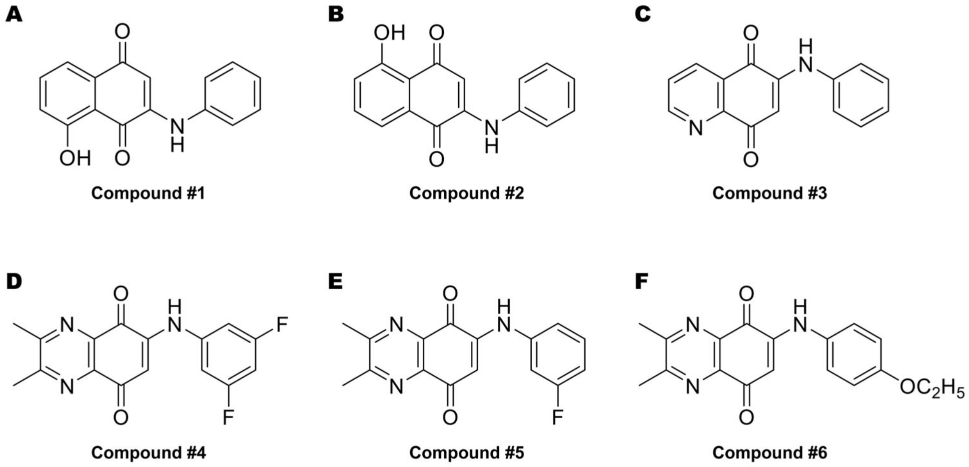


**Supplementary Figure S1. Structures of tested NQ-derived compounds**

(A-F) Chemical structures of NQ derivatives evaluated for anti-ferroptotic activity. (A) Compound #1 : 8-hydroxy-2-anilino-1,4-naphthoquinone (8-HANQ), (B) Compound #2 : 5-hydroxy-2-anilino-1,4-naphthoquinone (5-HANQ), (C) Compound #3 : 6-anilino-5,8-quinolinedione, (D) Compound #4 : 2,3-dimethyl-6-(3,5-difluoroanilino)-5,8-quinoxalinedione, (E) Compound #5 : 2,3-dimethyl-6-(3-fluoroanilino)-5,8-quinoxalinedione, (F) Compound #6 : 2,3-dimethyl-6-(4-ethoxyanilino)-5,8-quinoxalinedione.

Academic Editor(s): Avi Domb

Received: 29 September 2025
Revised: 28 October 2025
Accepted: 30 October 2025
Published: date

**Citation:** Lee, D.; Na, E.J.; Heo, Y.; Yu, J.; Kim, H.-J. 8-Hydroxy-2-Anilino-1,4-Naphthoquinone Prevents Against Ferroptotic Neuronal Death and Kainate-Induced Epileptic Seizures. *Pharmaceutics* **2025**, *17*, x. https://doi.org/10.3390/xxxxx

**Copyright:** © 2025 by the authors. Submitted for possible open access publication under the terms and conditions of the Creative Commons Attribution (CC BY) license (https://creativecommons.org/licenses/by/4.0/).

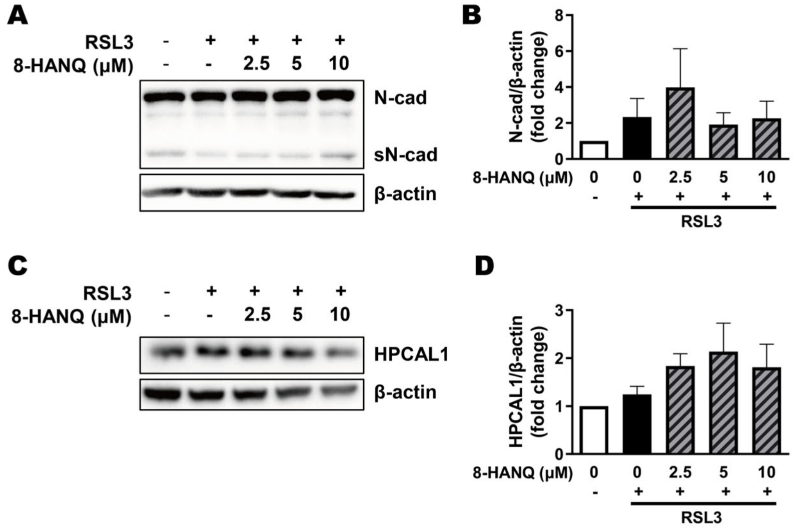


**Supplementary Figure S2**. Unchanged expression of full-length N-cadherin and hippocalcin-like 1 during neuronal ferroptosis (A-D) Western blot analysis and quantification of N-cadherin (N-cad; A-B, n=7) and hippocalcin-like 1 (HPCAL1; C-D, n=4) in HT22 cells. Cells were pretreated with 8-HANQ (2.5-10 μM) for 30 min, followed by exposure to 200 nM RSL3 for 24 h. Statistical significance was assessed using Student's t-test and data represent means ± SEM. n indicates independent experiments.

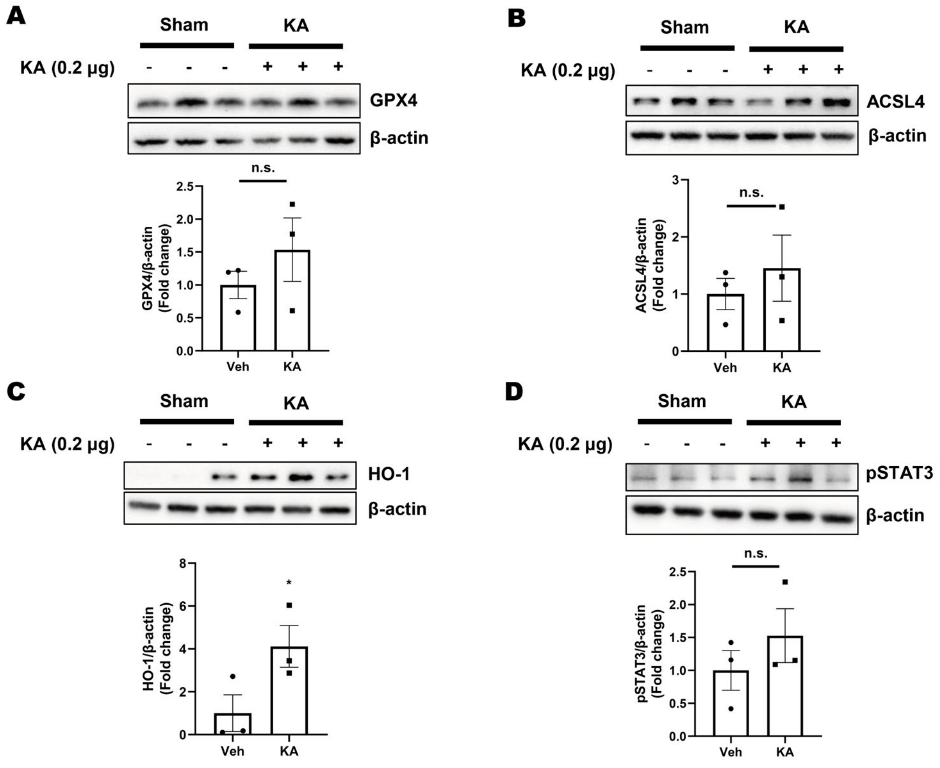


**Supplementary Figure S3.** Unaffected expression of GPX4, ACSL4, and pSTAT3, with induction of HO-1 after KA administration. (A-D) Western blot analysis and quantification of GPX4 (A), ACSL4 (B), HO-1 (C), and pSTAT3 (D) in hippocampal tissues collected 72 h after intracerebroventricular kainate (KA) administration. Mice received vehicle (Veh; 40% DMSO in saline, 1 μl) or 8-HANQ (1 μg) together with 0.2 μg KA via i.c.v. injection. Statistical significance was assessed using Student's t-test and data are presented as means ± SEM from 3 animals per group (*p<0.05 vs. Veh).

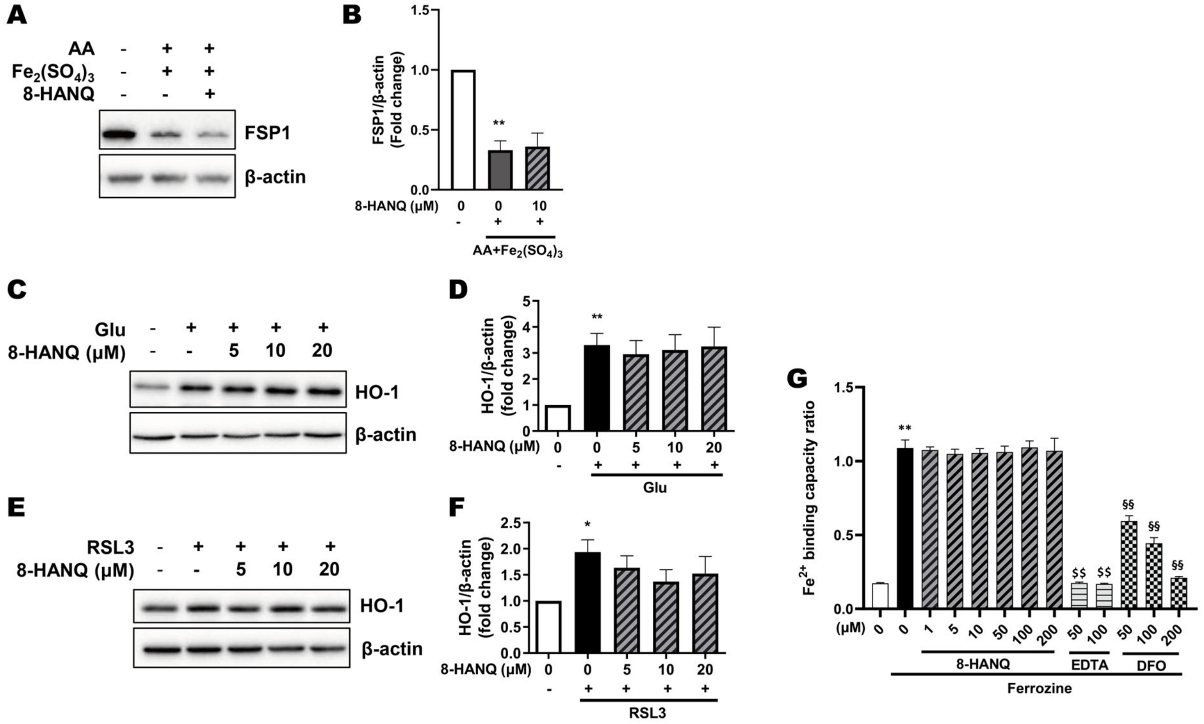


**Supplementary Figure S4.** Lack of involvement of FSP1, iron metabolism, and iron chelation in the neuroprotective anti-ferroptotic action of 8-HANQ. (A-F) Western blot analysis of ferroptosis-related proteins. The expression levels of FSP1 (A-B) and HO-1 (C-F) were evaluated in HT22 cells treated with 8-HANQ (5-20 μM). Ferroptosis was induced by a combination of 100 μM arachidonate (AA) and 100 μM iron (III) sulfate ($Fe_2(SO_4)_3$), 30 mM glutamate (Glu) or 200 nM RSL3 for 12 h (FSP1, n=4) or 24 h (HO-1; C-D, n=8; E-F, n=4). Statistical significance was determined by Student's t-test. (G) $Fe^{2+}$-chelating activity of 8-HANQ (1-200 μM) was assessed in a cell-free system. EDTA and deferoxamine (DFO) were used as positive controls (n=4). Statistical significance was determined by one-way ANOVA. Data are presented as means ± SEM. (**$p<0.01$ vs. Control (0); $$$p<0.01$ and §§$p<0.01$ vs. Ferrozine). n indicates independent experiments.
